# Supplementary figures and images for: An optimised protocol for the detection of lipofuscin, a versatile and quantifiable marker of cellular senescence
Source: PLoS One. 2024 Jul 15;19(7):e0306275. doi: 10.1371/journal.pone.0306275 (PMC11249248; doi:10.1371/journal.pone.0306275)

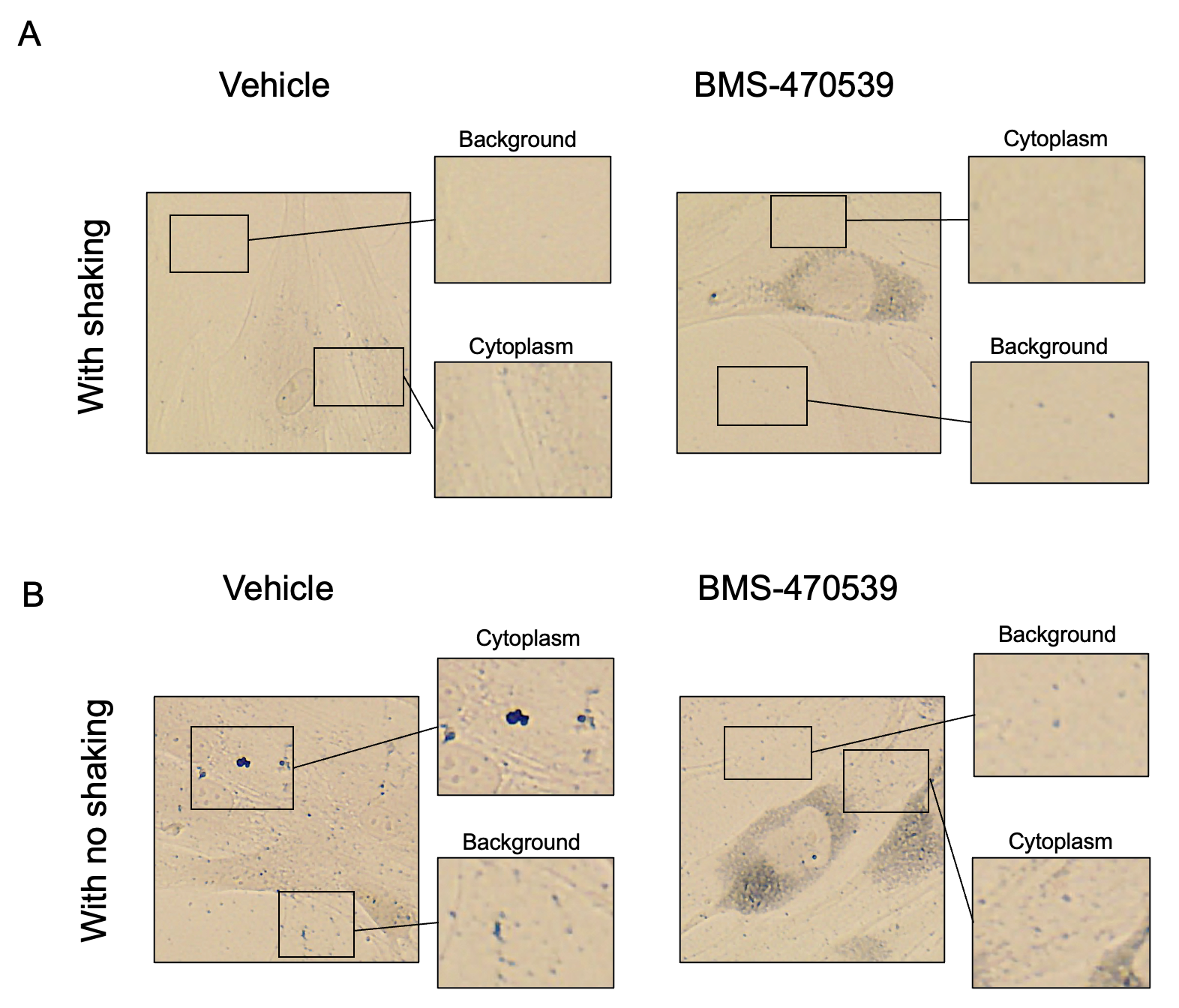

Supplement: S1 Fig — Human dermal fibroblasts were treated with vehicle (PBS) or 10μM BMS-470539 for 6 days and then stained with Sudan black B. The method was tested with (A) and without (B) using an orbital shaker during the incubation of the cells with the SBB solution. Images were captured using an EVOS XL Core Imaging System at 40X magnification. Selected areas were zoomed-in to highlight cytoplasmic and extracellular regions to determine the level of background and presence of SBB dye precipitates. (TIFF) [file pone.0306275.s001.tiff]

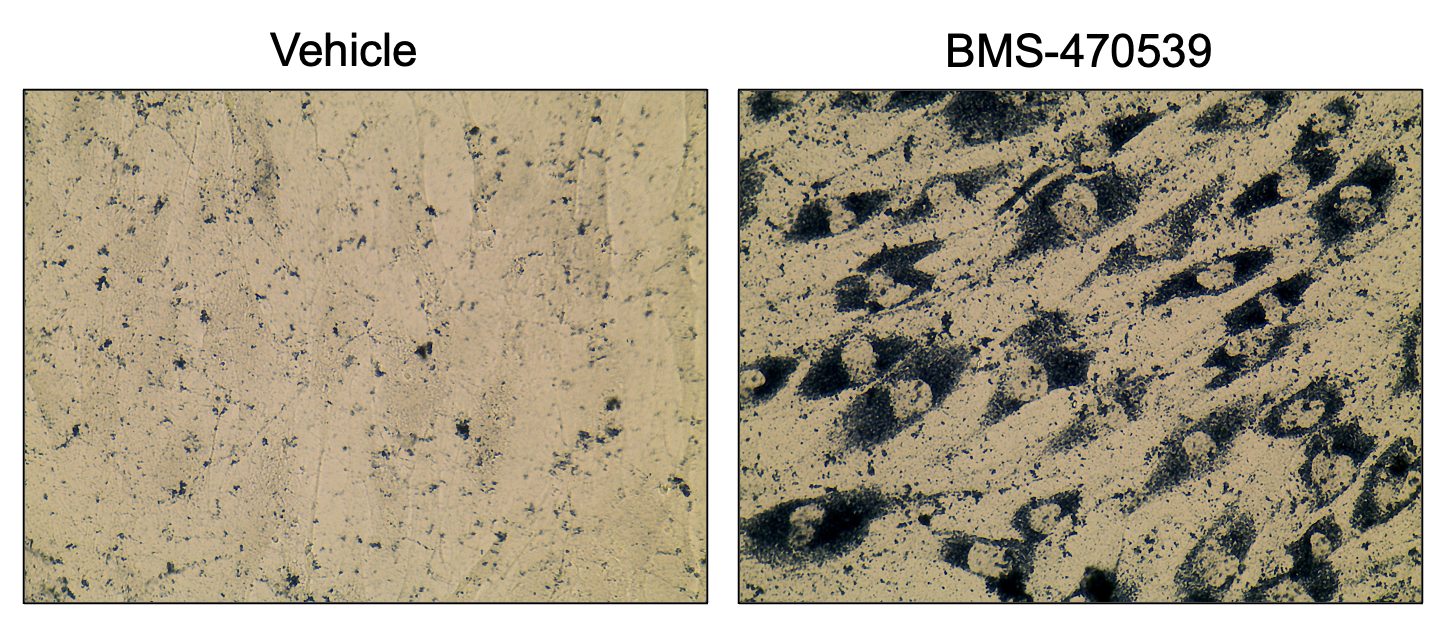

Supplement: S2 Fig — Human dermal fibroblasts were treated with vehicle (PBS) or 10μM BMS-470539 for 6 days and then stained with Sudan Black B using the protocol described herein. In this case, the Sudan black B solution used was prepared 7 days prior staining instead of freshly prepared the night before as indicated in the protocol. Images were captured using an EVOS XL Core Imaging System at 40X magnification. (TIFF) [file pone.0306275.s002.tiff]

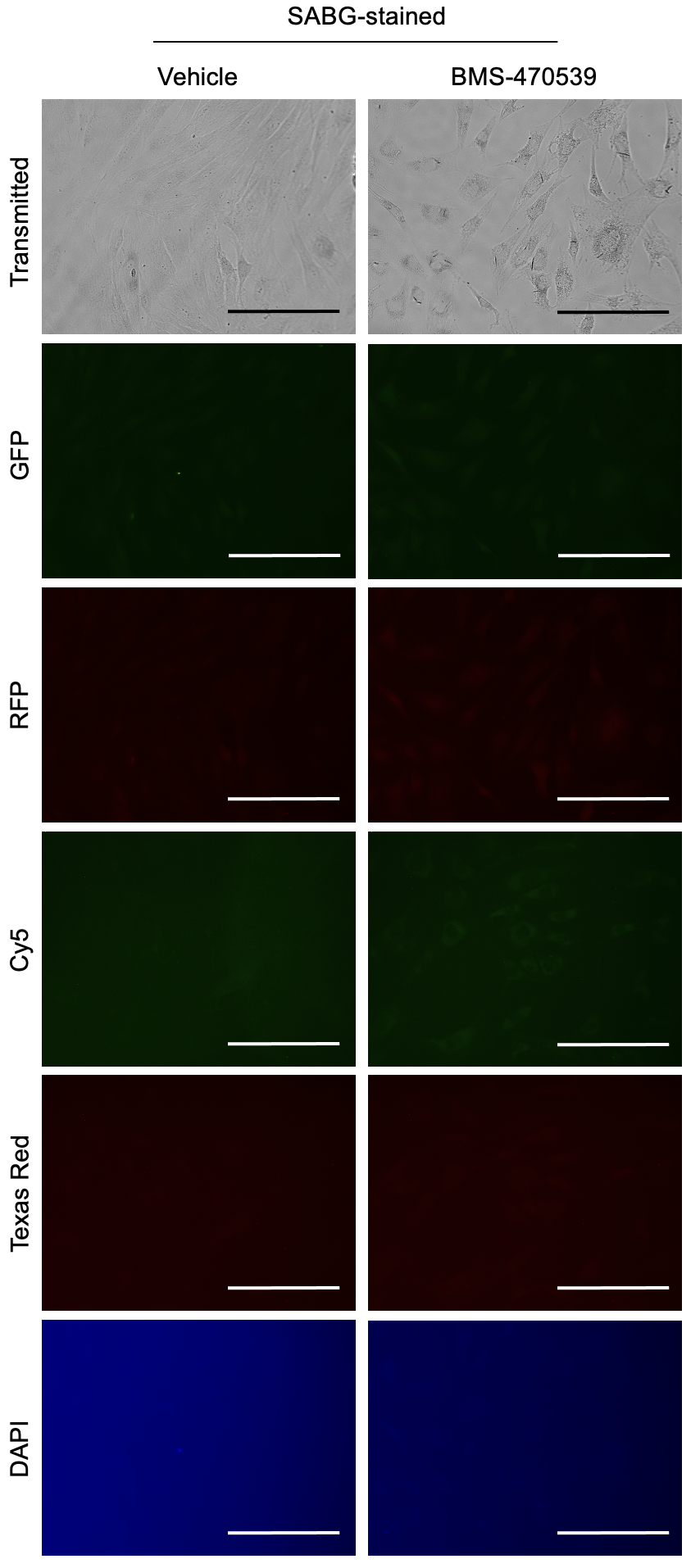

Supplement: S3 Fig — Human dermal fibroblasts were treated with vehicle (PBS) or 20μM BMS-470539 for 6 days and fluorescence signal determined at multiple channels before histochemical determination of SA-β-Gal activity. Images were captured using the EVOS FL Imaging System (ThermoFisher) with the following channels: GFP (green, EX470/22—EM525/50), RFP (orange EX531/40—EM593/40), Texas Red (red, EX585/29—EM628/32), Cy5 (far-red, EX628/40—EM685/40), DAPI (blue, EX357/44—EX447/60) and transmitted channels. Scale bars represent 200μm. (TIFF) [file pone.0306275.s003.tiff]

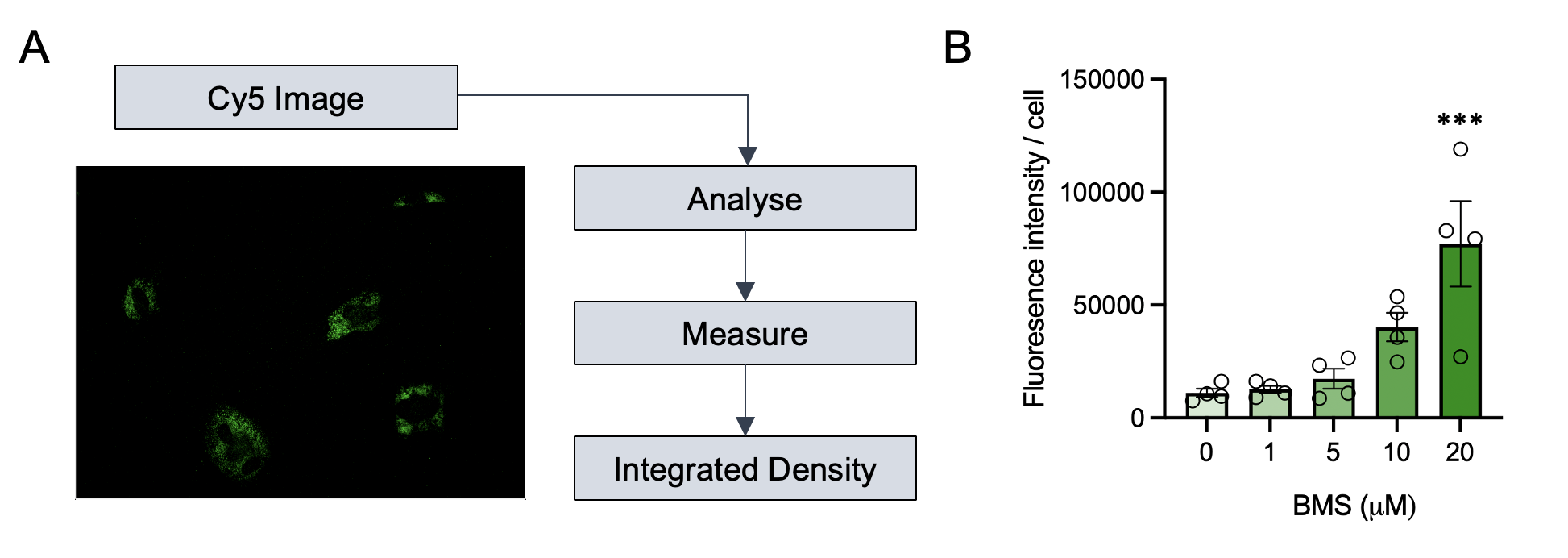

Supplement: S4 Fig — A) To calculate the fluorescence signal per cell in the far-red channel, Cy5 images were imported into Fiji and the integrated density measure function used to measure the integrated density of the whole image (Analyze → Measure→ Integrated density). This value was then normalised by the number of cells in the image to calculate the fluorescence per cell. B) Human dermal fibroblasts were treated with vehicle (PBS) or increasing concentrations of BMS-470539 for 6 days and subjected to the SBB staining protocol. The fluorescence intensity per image was calculated as explained in A, and normalised by the number of cells in each image. Data represent the mean ± SEM (n = 4; One-way ANOVA with multiple comparison correction vs. vehicle, ***p<0.001). (TIFF) [file pone.0306275.s004.tiff]
