# Supplementary material for: An optimised protocol for the detection of lipofuscin, a versatile and quantifiable marker of cellular senescence
Source: PLoS One. 2024 Jul 15;19(7):e0306275. doi: 10.1371/journal.pone.0306275 (PMC11249248; doi:10.1371/journal.pone.0306275)
Supplement: S1 File — This can also be accessed on protocols.io (https://dx.doi.org/10.17504/protocols.io.x54v9yw91g3e/v1). (PDF) [file pone.0306275.s005.pdf]

Jun 18, 2024

An optimised protocol for the detection of lipofuscin in cells cultured in vitro

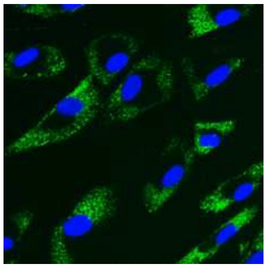

DOI
dx.doi.org/10.17504/protocols.io.x54v9yw91g3e/v1

Camilla SA Davan-Wetton[1], Trinidad Montero-Melendez[1]

[1]The William Harvey Research Institute, Queen Mary University of London, London EC1M 6BQ, United Kingdom

Montero-Melendez Group
The William Harvey Research Institute, Queen Mary University...

OPEN ACCESS

DOI: dx.doi.org/10.17504/protocols.io.x54v9yw91g3e/v1

Protocol Citation: Camilla SA Davan-Wetton, Trinidad Montero-Melendez 2024. An optimised protocol for the detection of lipofuscin in cells cultured in vitro. **protocols.io** https://dx.doi.org/10.17504/protocols.io.x54v9yw91g3e/v1

License: This is an open access protocol distributed under the terms of the **Creative Commons Attribution License**, which permits unrestricted use, distribution, and reproduction in any medium, provided the original author and source are credited

Protocol status: Working
We use this protocol and it's working

Created: May 27, 2022

Last Modified: June 18, 2024

Protocol Integer ID: 63346

Keywords: Senescence, lipofuscin, lysosomes, immunofluorescence, ageing, microscopy,

Funders Acknowledgement:
Barts Charity
Grant ID: G-002392
Biotechnology and Biological Sciences Research Council (BBSRC)
Grant ID: BB/M009513/1

Disclaimer

DISCLAIMER – FOR INFORMATIONAL PURPOSES ONLY; USE AT YOUR OWN RISK
The protocol content here is for informational purposes only and does not constitute legal, medical, clinical, or safety advice, or otherwise. Information presented in this protocol should not substitute for independent professional judgment, advice, diagnosis, or treatment. Any action you take or refrain from taking using or relying upon the information presented here is strictly at your own risk. You agree that neither the Company nor any of the authors, contributors, administrators, or anyone else associated with **protocols.io**, can be held responsible for your use of the information contained in or linked to this protocol or any of our Sites/Apps and Services.

Abstract

Lipofuscin is a complex material that accumulates in lysosomes and it is considered a marker of cellular senescence and ageing. This substance can be detected using the lipophilic stain Sudan Black B. This protocol describes an easy, straightforward and inexpensive method for the staining of lipofuscin using Sudan Black B and imaging via brightfield microscopy. Additionally, this protocol offers an alternative method for detection of Sudan Black B stained lipofuscin using fluorescence microscopy, which facilitates co-staining of Sudan Black B with conventional antibody-based immunofluorescence staining techniques.

Image Attribution

Image credit: Camilla SA Davan-Wetton

Guidelines

This protocol should be performed following standard laboratory practices, including the wearing of suitable PPE like laboratory coat, gloves and goggles.

Materials

Reagents and Solutions

- 4% Paraformaldehyde solution
- Sudan Black B **Merck MilliporeSigma (Sigma-Aldrich)**
- 70% Ethanol
- Aluminium sulphate **Merck MilliporeSigma (Sigma-Aldrich)**
- Phosphate-buffered saline
- Nuclear Fast Red **Merck MilliporeSigma (Sigma-Aldrich)**
- 4′,6-diamidino-2-phenylindole (DAPI) **Merck MilliporeSigma (Sigma-Aldrich)**

Materials

- Cell strainer pore size 70µm
- PDVF syringe filter pore size 0.45µm
- PDVF syringe filter pore size 0.22µm
- 20ml syringes
- Magnetic stirring bars

Equipment

Equipment	
**Magnetic Stirrer, Lab Disc**	NAME
Magnetic Stirrer	TYPE
VWR	BRAND
442-0883	SKU

Equipment	
**FTA-1**	NAME
Aspirator with trap flask	TYPE
Biosan	BRAND
BS-040108-AAK	SKU

Equipment	
**PMS-1000i**	NAME
Microplate Shaker	TYPE
Grant Bio	BRAND
SHA7910	SKU

Equipment	
**EVOS XL Core Imaging System**	NAME
Bright field microscope	TYPE
ThermoFisher Scientific	BRAND
AMEX1000	SKU

Equipment

**EVOS® FL Auto Imaging System**	NAME
Fluorescence microscope	TYPE
ThermoFisher Scientific	BRAND
AMEX1000	SKU
Equipped with Cy5 (628/40 nm Excitation; 692/40 nm Emission) and DAPI (357/44 nm Excitation; 447/60 nm Emission) Light Cubes	SPECIFICATIONS

Software

- Image processing package Fiji (ImageJ2, version 2.14.0/1.54f)

Safety warnings

It is recommended to familiarise yourself with the safety data sheets (SDS) relevant to this method and to conduct the corresponding risk assessment before commencing the protocol. For chemical hazards, follow appropriate safety precautions and waste disposal methods as per the local guidelines and regulations in your area.

Before start

This protocol is optimised for the staining of cells cultured in vitro in 24-well plates. Plate your cells and treat with appropriate compounds or stimuli for the required time. Remember to prepare the Saturated Sudan black B solution (steps 1-2) the day before you plan to stain your cells, as this solution requires to be stirred overnight for complete dissolution.

Saturated Sudan Black B solution preparation

16h

1 Dissolve 1.2 g Sudan Black B in 80 mL 70% ethanol in a glass bottle and stir Overnight on a magnetic stirrer.

2 Before use, filter the solution three times as follows:
- first through a **70µm** cell strainer
- then through a **0.45µm** syringe filter
- finally through a **0.22µm** syringe filter.

Note

Prepare Sudan Black solution fresh. Storing and re-using old solutions is not recommended.

Nuclear Fast Red solution preparation

3 Prepare a 5% aluminium sulphate solution by dissolving 25 g aluminium sulphate in 500 mL of distilled water. Stir until dissolved, sterilise by filtration using a 0.22µm filter and store at Room temperature .

Note

Sterilisation is recommended when preparing a larger volume of aluminium sulphate to prevent microorganism growth, as a 500ml batch can last for several months.

4 Dissolve 200 mg Nuclear Fast Red in 200 mL boiling 5% aluminium sulphate solution. Boil for 00:05:00 and allow it to return to Room temperature before use. 5m

Sudan Black B staining

5 Remove cell culture medium and wash cells once in PBS.

Note

For this and subsequenct steps where solutions are removed, an aspirator with a trap flask can be used.

6 Fix cells in 4% PFA for 00:15:00 at Room temperature . 15m

Note

PFA is a chemical hazard and needs to be used inside a chemical fume hood.

7 Remove PFA solution and incubate cells in 70% ethanol for 00:02:00 . 2m

Note

PFA is a chemical hazard and needs to be disposed of in an appropriate waste container.

8 iunspecificRemove ethanol and incubate cells in the freshly prepared, triple filtered Sudan Black B solution for 00:08:00 . Place plate on a plate shaker at 200 rpm for the duration of the staining. 8m

Note

Shaking the plate during the staining process is strongly advised to avoid SBB precipitates which will produce unspecific staining.

9 Remove Sudan Black B solution and wash the cells in distilled water for 00:05:00 , replacing the plate on the plate shaker at 200 rpm for the duration of the washing. 5m

Note

Sudan Black B solution should be disposed of according to local waste management regulations and should not enter the drainage system.

At this stage, the protocol is subdivided depending if qualitative (A) or quantitative (B) staining is desired.

A) Counterstaining with Nuclear Fast Red solution

10 Remove the distilled water and incubate cells in the Nuclear Fast Red solution for 00:10:00 . Place the plate on the plate shaker at 200 rpm for the duration of the staining. 10m

11 Remove the Nuclear Fast Red solution and wash wells in PBS for 00:10:00 , replacing the plate on the plate shaker at 200 rpm for the duration of the washing. 10m

12 Replace the PBS with fresh PBS and visualise cells using a brightfield microscope.

B) Quantification of Sudan Black B staining

20m

13 Remove the distilled water and incubate cells in 1 ug/ml DAPI solution for 00:10:00 . 10m

Note

The plate should be protected from light by covering with aluminium foil from now onwards as DAPI is light sensitive.

14 Remove the DAPI solution and wash the cells once in PBS for 00:10:00 , replacing the plate on the plate shaker at 200 rpm for the duration of the washing. 10m

15 Replace the PBS with fresh PBS and visualise cells using a fluorescence microscope. Sudan Black B can be visualised with a Cy5 filter (far-red, 628/40 nm Excitation; 692/40 nm Emission).

16 Quantification may be performed, either by calculating the proportion of fluorescent cells (i.e. percentage of positive cells), or by measuring the total fluorescence intensity per cell.

Note

Total fluorescence can be quantified in Fiji using the 'Integrated Density' measurement.
